# Supplementary material for: Antioxidant nanozyme counteracts HIV‐1 by modulating intracellular redox potential
Source: EMBO Mol Med. 2021 Apr 1;13(5):e13314. doi: 10.15252/emmm.202013314 (PMC8103102; doi:10.15252/emmm.202013314)
Supplement: Supplementary file 1 — Appendix [file EMMM-13-e13314-s003.pdf]

Antioxidant nanozyme counteracts HIV-1 by modulating intracellular redox potential

Shalini Singh[1, 2], Sourav Ghosh[3], Virender Kumar Pal[1, 2], MohamedHusen Munshi[2], Pooja Shekhar[4], Diwakar Tumkur Narasimha Murthy[5], Govindasamy Mugesh[3*], and Amit Singh[1, 2*]

[1]Department of Microbiology and Cell Biology, Indian Institute of Science, Bangalore-560012, India

[2]Centre for Infectious Disease Research (CIDR), Indian Institute of Science, Bangalore 560012, India

[3]Department of Inorganic and Physical Chemistry, Indian Institute of Science, Bangalore 560012, India.

[4]Bangalore Medical College and Research Institute, Bangalore – 560002

[5]Department of Internal Medicine, Bangalore Medical College and Research Institute, Bangalore – 560002

*** Correspondence:**

Amit Singh (asingh@iisc.ac.in) and Govindasamy Mugesh (mugesh@iisc.ac.in)

Running Title - Anti-HIV potential of V_2O_5-nanosheets

Supplementary Material (SI Appendix)

Appendix Fig S1. Survival assay for Vs-treated J1.1 cells.

Appendix Fig S2. XRD pattern and TEM of Vs

Appendix Fig S3. GR- coupled assay for Vs under varied conditions.

Appendix Fig S4. Preparation of Stable cell lines.

Appendix Fig S5. Time dependent induction of Selenium (Se) starvation-mediated oxidative stress.

Appendix Fig S6. HIV inhibitory activity of Vs is reversible.

Appendix Fig S7. Expression of antioxidant genes in primary $CD4^{+}$ T cells are lowered upon Vs exposure.

Appendix Fig S8. Viability of primary CD4+ T cells is not affected by Vs treatment.

Appendix Table S1. List of Genes and probes used for nCounter Gene Expression analysis

Appendix Table S2. Normalized intensity values of differentially expressed gene in nCounter Gene Expression Assay

Appendix Table S3. Fold change ($FC>1.5$, $p<0.05$) of differentially expressed gene in nCounter Gene Expression Assay

Appendix Table S4. Details of HIV-infected subjects used in the study.

Appendix Table S5. List of primers used in the study.

Appendix Figures

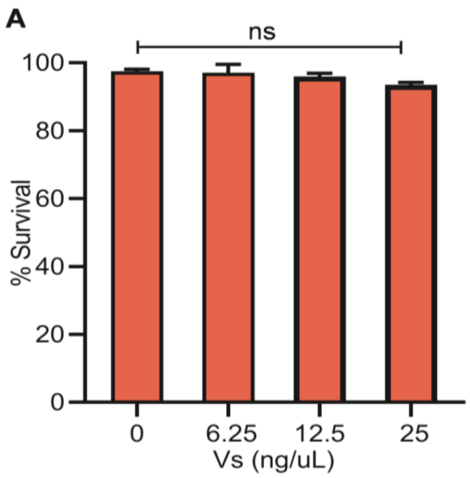

Appendix Fig S1. Survival assay for Vs-treated J1.1 cells.

A. J1.1 cells were treated with increasing concentrations of Vs – 6.25 to 25 ng/µL- for 15 min and cell survival was analysed by flow cytometry after 24 h by propidium iodide (PI) staining. Data are representative of two independent experiments done in duplicate (mean ± SEM). ns - non-significant by Student’s *t* test.

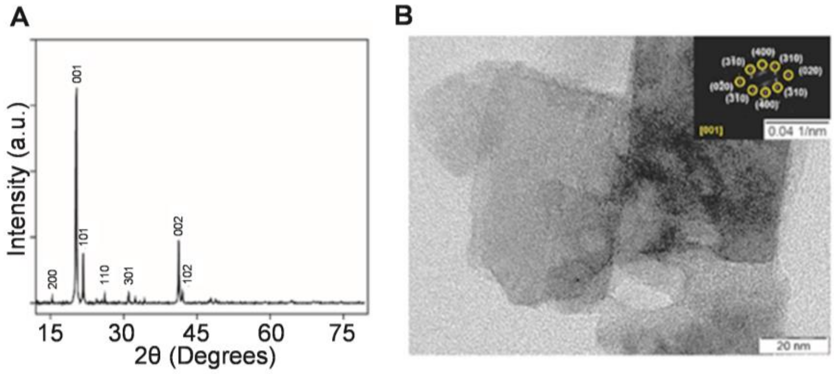

Appendix Fig S2. XRD pattern and TEM of Vs.

A. Powder XRD of Vs where all the diffraction peaks were indexed to standard V_2O_5 orthorhombic phase (JCPDS = 41-1426, space group Pmmn).

B. Transmission electron microscopy (TEM) image of Vs thin nanosheets (scale – 20 nm) and selected area electron diffraction (SAED) (inset) shows the crystalline nature of the material.

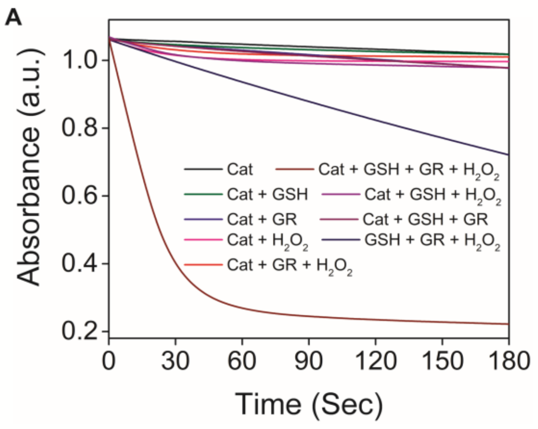

Appendix Fig S3. GR- coupled assay for Vs under varied conditions.

A. The decrease in absorbance of NADPH (0.2 mM under different assay conditions were monitored during GR coupled assay.

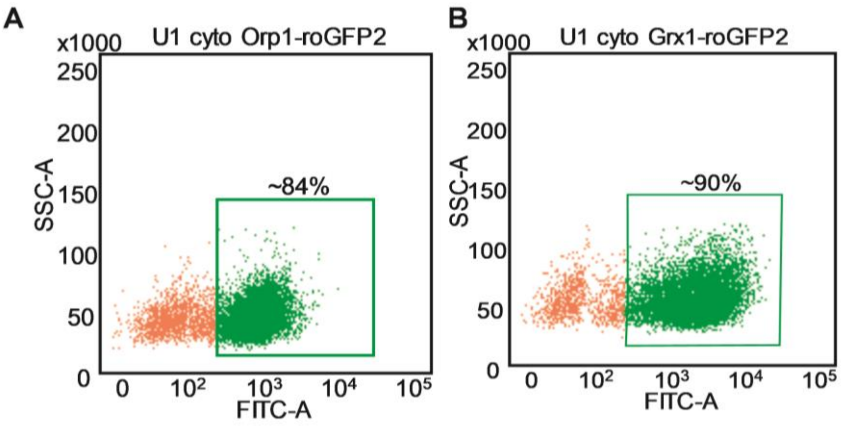

Appendix Fig S4. Preparation of Stable cell lines.

A, B. Dot plot validating the stable expression of the biosensors in **(A)** U1 cyto-Orp1-roGFP2 and **(B)** U1 cyto-Grx1-roGFP2 cell lines.

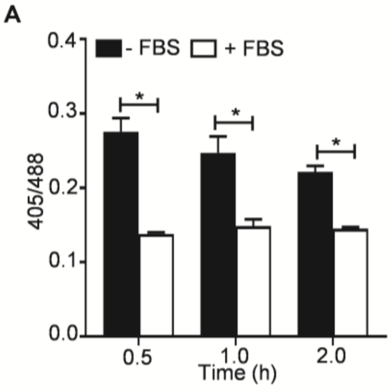

Appendix Fig S5. Time dependent induction of Selenium (Se) starvation-mediated oxidative stress.

A. U1-Grx1-roGFP2 cells were cultured in serum free medium (to deplete Se) for various time periods and biosensor response was measured by flow cytometry. Data are representative of two experiments done in duplicates (mean ± SEM). * $P<0.05$, by Mann Whitney test.

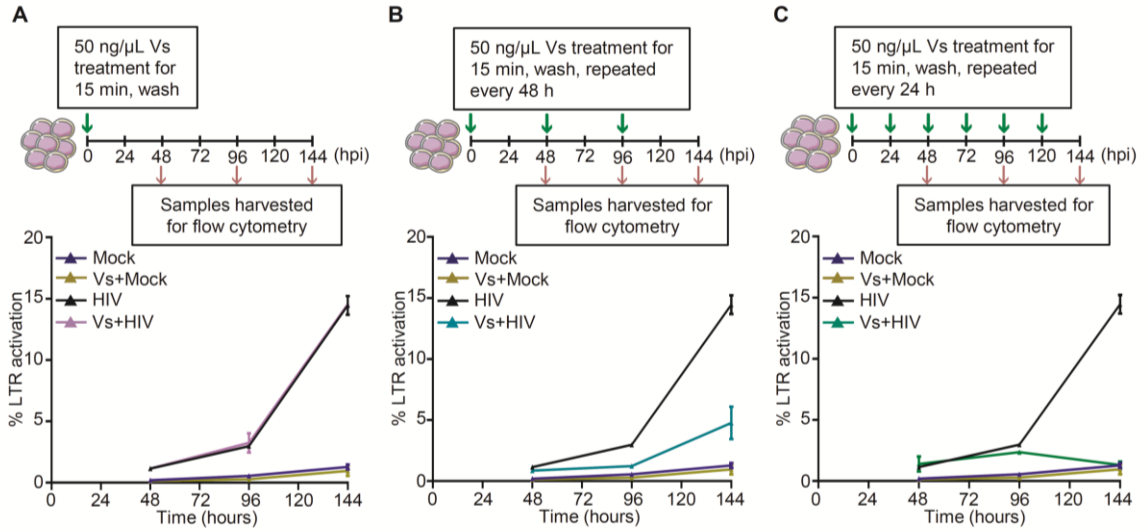

Appendix Fig S6. HIV inhibitory activity of Vs is reversible.

A-C. CEM-GFP cells pre-treated with 50 ng/µL of Vs were infected with 0.1 moi of HIV-NL4.3. Vs treatment was continued for different time points during the experiment - (A) single dose (pre-treatment only), (B) treatment every 48 h and (C) treatment every 24 h. EGFP fluorescence was analysed by flow cytometry at 2, 4, and 6 dpi. Experiment was performed once in duplicate (mean ± SD).

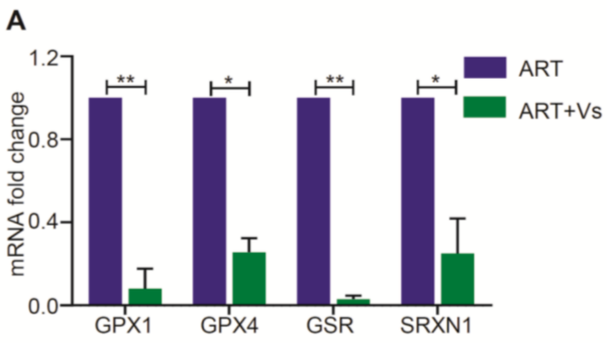

Appendix Fig S7. Expression of antioxidant genes in primary $CD4^+$ T cells are lowered upon Vs exposure.

A. ARV or Vs + ARVs treated cells were cultured for 21 days under standard conditions (*see Materials and* Methods). 21 days post culturing, RNA was isolated and expression of antioxidant genes, GPX1, GPX4, GSR, and SRXN1 were analysed by RT-qPCR. Data are representative of three donors. ** $P<0.01$, * $P<0.05$, ns – non-significant by 2-way ANOVA with Sidak's multiple comparison.

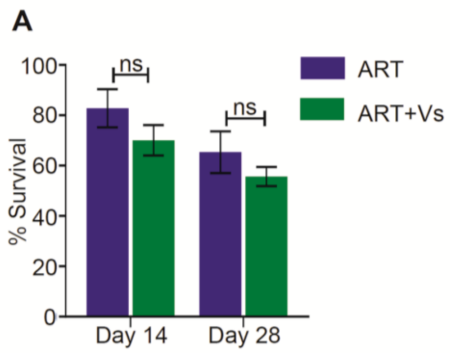

Appendix Fig 8. Viability of primary CD4$^+$ T cells is not affected by Vs treatment.

A. Primary CD4$^+$ T cells isolated from ARV-suppressed individuals were culture in presence of ARV alone or Vs + ARV (*as mentioned in Materials and Methods*). At day 14 and day 28 ARV and ARV+Vs treatment, cells were stained with Aqua Dead Cell staining dye and cell viability was analysed using flow cytometry. Data are representative of three donors. ns – non-significant by 2-way ANOVA with Sidak's multiple comparison.

Appendix Table

Appendix Table S1. List of Genes and probes used for nCounter Gene Expression analysis.

Gene Name	Target sequence
ABCF1	GATGTCCTCCCGCCAAGCCATGTTAGAAAATGCATCTGACATCAAGCTGGAGAAGTTCAGCATCTCCGCTCATGGCAAGGAGCTGTTCGTCAATGCAGAC
ACTB	TGCAGAAGGAGATCACTGCCCTGGCACCCAGCACAATGAAGATCAAGATCATTGCTCCTCCTGAGCGCAAGTACTCCGTGTGGATCGGCGGCTCCATCCT
AHCY	ATCCTTGGCCGGCACTTTGAGCAGATGAAGGATGATGCCATTGTGTGTAACATTGGACACTTTGACGTGGAGATCGATGTCAAGTGGCTCAACGAGAACG
AKR1C2	CTGCAGAGGTTCCTAAAAGTAAAGCTCTAGAGGCCGTCAAATTGGCAATAGAAGCCGGGTTCCACCATATTGATTCTGCACATGTTTACAATAATGAGGA
ALB	TCTTACCAAAGTCCACACGGAATGCTGCCATGGAGATCTGCTTGAATGTGCTGATGACAGGGCGGACCTTGCCAAGTATATCTGTGAAAATCAAGATTCG
ALOX12	TTACAGCCCGGAATGAGCAACTTGACTGGCCCTATGAATATCTGAAGCCCAGCTGCATAGAGAACAGTGTCACCATCTGAGCCCTAGAGTGACTCTACCT
AOX1	GCGATAGTCAATTCAGGAATGAGAGTCTTTTTTGGAGAAGGGGATGGCATTATTAGAGAGTTATGCATCTCATATGGAGGCGTTGGTCCAGCCACCATCT
APOBEC3G	GAAAAGAGACGGTCCGCGTGCCACCATGAAGATCATGAATTATGACGAATTTCAGCACTGTTGGAGCAAGTTCGTGTACAGCCAAAGAGAGCTATTTGAG
APOE	GGGCTGCGTTGCTGGTCACATTCCTGGCAGGATGCCAGGCCAAGGTGGAGCAAGCGGTGGAGACAGAGCCGGAGCCCGAGCTGCGCCAGCAGACCGAGTG
ATOX1	GCTGAAGCTGTCTCTCGGGTCCTCAATAAGCTTGGAGGAGTTAAGTATGACATTGACCTGCCCAACAAGAAGGTCTGCATTGAATCTGAGCACAGCATGG
B2M	CGGGCATTCCTGAAGCTGACAGCATTCGGGCCGAGATGTCTCGCTCCGTGGCCTTAGCTGTGCTCGCGCTACTCTCTCTTTCTGGCCTGGAGGCTATCCA
BAD	AGGGAGGGCTGACCCAGATTCCCTTCCGGTGCGTGTGAAGCCACGGAAGGCTTGGTCCCATCGGAAGTTTTGGGTTTTCCGCCCACAGCCGCCGGAAGTG
BAG2	AAGAGTCATTTAATGTCGCTCTACAGTGCATGTTCATCTGAGGTGCCACATGGGCCAGTTGATCAGAAGTTTCAATCCATAGTAATTGGCTGTGCTCTTG
BANF1	GGAGGAAAGGGGTTTTGACAAGGCCTATGTTGTCCTTGGCCAGTTTCTGGTGCTAAAGAAAGATGAAGACCTCTTCCGGGAATGGCTGAAAGACACTTGT
BAX	TTTTTCCGAGTGGCAGCTGACATGTTTTCTGACGGCAACTTCAACTGGGGCCGGGTTGTCGCCCTTTTCTACTTTGCCAGCAAACTGGTGCTCAAGGCCC
BCL2	GTGAAGCAGAAGTCTGGGAATCGATCTGGAAATCCTCCTAATTTTTACTCCCTCTCCCCGCGACTCCTGATTCATTGGGAAGTTTCAAATCAGCTATAAC
BHMT	CGCAGCGGGAAGGCTCGCCTAGTCGGTCCGCATCCGTGTCGACCACCTGTCTGGACACCACGAAGATGCCACCCGTTGGGGGCAAAAAGGCCAAGAAGGG
BNIP3	AGGGGGCATATTCTCTGCAGAATTTCTGAAAGTTTTCCTTCCATCTCTGCTGCTCTCTCATTTGCTGGCCATCGGATTGGGGATCTATATTGGAAGGCGT

BTRC	ACACCTACATCTCCAGATAAATAACCATACACTGACCTCATACTTGCCCAGGACCCATTAAAGTTGCGGTATTTAACGTATCTGCCAATACCAG GATGAG
CASP3	ACTCCACAGCACCTGGTTATTATTCTTGGCGAAATTCAAAGGATGGCTCCTGGTTCATCCAGTCGCTTTGTGCCATGCTGAAACAGTATGCCGA CAAGCT
CASP8	AGATGGACTTCAGCAGAAATCTTTATGATATTGGGGAACAACTGGACAGTGAAGATCTGGCCTCCCTCAAGTTCCTGAGCCTGGACTACATTCC GCAAAG
CAT	ATGCTTCAGGGCCGCCTTTTTGCCTATCCTGACACTCACCGCCATCGCCTGGGACCCAATTATCTTCATATACCTGTGAACTGTCCCTACCGTGC TCGAG
CBS	GCGGGAGCGTGAAGGACCGCATCAGCCTGCGGATGATTGAGGATGCTGAGCGCGACGGGACGCTGAAGCCCGGGGACACGATTATCGAGCCG ACATCCGG
CBX5	TATGAAGAGAGACTGACATGGCATGCATATCCTGAGGATGCGGAAAACAAAGAGAAAGAAACAGCAAAGAGCTAAAGGAGGGGATGGTCTC TGTCATTTC
CCL2	GAGGAACCGAGAGGCTGAGACTAACCCAGAAACATCCAATTCTCAAACTGAAGCTCGCACTCTCGCCTCCAGCATGAAAGTCTCTGCCGCCCT TCTGTGC
CCL3	CTGTGTAGGCAGTCATGGCACCAAAGCCACCAGACTGACAAATGTGTATCGGATGCTTTTGTTCAGGGCTGTGATCGGCCTGGGGAAATAATA AAGATGC
CCL4	GAAGCTTCCTCGCAACTTTGTGGTAGATTACTATGAGACCAGCAGCCTCTGCTCCCAGCCAGCTGTGGTATTCCAAACCAAAAGAAGCAAGCA AGTCTGT
CCL5	AGTGTGTGCCAACCCAGAGAAGAAATGGGTTCGGGAGTACATCAACTCTTTGGAGATGAGCTAGGATGGAGAGTCCTTGAACCTGAACTTACA CAAATTT
CCL8	AAGGAGAGATGGGTCAGGGATTCCATGAAGCATCTGGACCAAATATTTCAAAATCTGAAGCCATGAGCCTTCATACATGGACTGAGAGTCAGA GCTTGAA
CCNT1	GCACAAGACTCACCCATCTAATCATCATCATCATCATAATCACCACTCACACAAGCACTCTCATTCCCAACTTCCAGTTGGTACTGGGAACAAA CGTCCT
CCR2	TCTGATCTGCTTTTTCTTATTACTCTCCCATTGTGGGCTCACTCTGCTGCAAATGAGTGGGTCTTTGGGAATGCAATGTGCAAATTATTCACAGG GCTGT
CCR3	CAGTGCTCTTTACCCAGAGGATACAGTATATAGCTGGAGGCATTTCCACACTCTGAGAATGACCATCTTCTGTCTCGTTCTCCCTCTGCTCGTTA TGGCC
CCR4	TCCCTTCCTGGCTTTCTGTTCAGCACTTGTTATACTGAGCGCAACCATACCTACTGCAAAACCAAGTACTCTCTCAACTCCACGACGTGGAAGG TTCTCA
CCR5	TAGGAACATACTTCAGCTCACACATGAGATCTAGGTGAGGATTGATTACCTAGTAGTCATTTCATGGGTTGTTGGGAGGATTCTATGAGGCAAC CACAGG
CCS	GGGTCCAGAATGGCTTCGGATTCGGGGAACCAGGGGACCCTCTGCACGTTGGAGTTCGCGGTGCAGATGACCTGTCAGAGCTGTGTGGACGCG GTGCGCA
CD209	TAGAGCTTGTTTTTCTGGCCCATCCTTGGAGCTTTATGAGTGAGCTGGTGTGGGATGCCTTTGGGGGTGGACTTGTGTTCCAAGAATCCACTCTC TCTTC
CD247	TGGCAGGACAGGAAAAACCCGTCAATGTACTAGGATACTGCTGCGTCATTACAGGGCACAGGCCATGGATGGAAAACGCTCTCTGCTCTGCTT TTTTTCT

CD4	TGGCAGGCGGAGAGGGCTTCCTCCTCCAAGTCTTGGATCACCTTTGACCTGAAGAACAAGGAAGTGTCTGTAAAACGGGTTACCCAGGACCCTAAGCTCC
CD44	ACACCATGGACAAGTTTTGGTGGCACGCAGCCTGGGGACTCTGCCTCGTGCCGCTGAGCCTGGCGCAGATCGATTTGAATATAACCTGCCGCTTTGCAGG
CD69	AGGACATGAACTTTCTAAAACGATACGCAGGTAGAGAGGAACACTGGGTTGGACTGAAAAAGGAACCTGGTCACCCATGGAAGTGGTCAAATGGCAAAGA
CD74	TTCAGCCCCCAGCCCCTCCCCCATCTCCCACCCTGTACCTCATCCCATGAGACCCTGGTGCCTGGCTCTTTCGTCACCCTTGGACAAGACAAACCAAGTC
CDK7	CTGAGGAACAGTGGCCGGACATGTGTAGTCTTCCAGATTATGTGACATTTAAGAGTTTCCCTGGAATACCTTTGCATCACATCTTCAGTGCAGCAGGAGA
CDK9	GGTGTTCGACTTCTGCGAGCATGACCTTGCTGGGCTGTTGAGCAATGTTTTGGTCAAGTTCACGCTGTCTGAGATCAAGAGGGTGATGCAGATGCTGCTT
CDKN1A	CATGTGTCCTGGTTCCCGTTTCTCCACCTAGACTGTAAACCTCTCGAGGGCAGGGACCACACCCTGTACTGTTCTGTGTCTTTCACAGCTCCTCCCACAA
CDO1	TGAATAATAGAGGAACTTACGGCACTCTGCCATTTGTTAATGAAAGGAAGTGCAGAGGATTTAGAAAAGTACATGATCCCCAGACCACAACAAACCAAAA
CEBPB	CAACCGCACATGCAGATGGGGCTCCCGCCCGTGGTGTTATTTAAAGAAGAAACGTCTATGTGTACAGATGAATGATAAACTCTCTGCTTCTCCCTCTGCC
COPS6	CAGATTTTTATGATCAATGCAACGACGTGGGGCTCATGGCCTACCTCGGCACCATCACCAAAACGTGCAACACCATGAACCAGTTTGTGAACAAGTTCAA
CR2	GGTGTCAAGCAAATAATATGTGGGGGCCGACACGACTACCAACCTGTGTAAGTGTTTTCCCTCTCGAGTGTCCAGCACTTCCTATGATCCACAATGGACA
CSAD	CTGCACTGACCTGTGCTGATATGGACTTCCTCCTCAACGAGCTGGAGCGGCTAGGCCAGGACCTGTGAGCCTTCTCTGTCTTGCTGCCGGCCTTGATACC
CTH	CCTCTGCAATCGAGGTCTGAAGACTCTACATGTCCGAATGGAAAAGCATTTCAAAAACGGAATGGCAGTTGCCCAGTTCCTGGAATCTAATCCTTGGGTA
CX3CL1	CCCCGGAGCTGTGGTAGTAATTCATATGTCCTGGTGCCCGTGTGAACTCCTCTGGCCTGTGTCTAGTTGTTTGATTCAGACAGCTGCCTGGGATCCCTCA
CXCL12	CCGCCCGCCCGCCCGCCCGCGCCATGAACGCCAAGGTCGTGGTCGTGCTGGTCCTCGTGCTGACCGCGCTCTGCCTCAGCGACGGGAAGCCCGTCAGCCT
CXCL8	ACAGCAGAGCACACAAGCTTCTAGGACAAGAGCCAGGAAGAAACCACCGGAAGGAACCATCTCACTGTGTGTAAACATGACTTCCAAGCTGGCCGTGGCT
CXCR4	ATTGATGTGTGTCTAGGCAGGACCTGTGGCCAAGTTCTTAGTTGCTGTATGTCTCGTGGTAGGACTGTAGAAAAGGGAACTGAACATTCCAGAGCGTGTA
CYBB	TTTGAAGCATGAAAAAAGAGGGTTGGAGGTGGAGAATTAACCTCCTGCCATGACTCTGGCTCATCTAGTCCTGCTCCTTGTGCTATAAAATAAATGCAGA
CYGB	AAGCACAAGGTGGAACCGGTGTACTTCAAGATCCTCTCTGGGGTCATTCTGGAGGTGGTCGCCGAGGAATTTGCCAGTGACTTCCCACCTGAGACGCAGA

DHCR24	CAGAGCCCAGCAAGCTGAATAGCATTGGCAATTACTACAAGCCGTGGTTCTTTAAGCATGTGGAGAACTATCTGAAGACAAACCGAGAGGGCCTGGAGTA
DUOX1	TGAGCCCACACCTCACCTCTGTTCTTCCTATTTCTGGCTGCCTCAGCCTTCTCTGATTTCCCACCTCCCAACCTTGTTCCAGGTGGCCATAGTCAGTCAC
DUOX2	AGGCACATCGTGTGTGTGGCAATCTTCTCGGCCATCTGTGTTGGCGTGTTTGCAGATCGTGCTTACTACTATGGCTTTGCCTCGCCACCCTCGGACATTG
DUSP1	TCAAGAATGCTGGAGGAAGGGTGTTTGTCCACTGCCAGGCAGGCATTTCCCGGTCAGCCACCATCTGCCTTGCTTACCTTATGAGGACTAATCGAGTCAA
ELANE	ACTTCTGCGGCGCCACCCTGATTGCGCCCAACTTCGTCATGTCGGCCGCGCACTGCGTGGCGAATGTAAACGTCCGCGCGGTGCGGGTGGTCCTGGGAGC
EP300	CCAGCCAGGCCCAACAGAGCAGTCCTGGATTAGGTTTGATAAATAGCATGGTCAAAAGCCCAATGACACAGGCAGGCTTGACTTCTCCCAACATGGGGAT
EPHX2	AGGAAGTGCTCCGAGACCGCTAAAGTCTGCCTCCCCAAGAATTTCTCCATAAAAGAAATCTTTGACAAGGCGATTTCAGCCAGAAAGATCAACCGCCCCA
EPX	GACAGGTTCTGGTGGCAGAAACGAGGTGTTTTCACCAAAAGACAGCGCAAGGCCCTGAGCAGAATTTCCTTGTCTCGAATTATATGTGACAATACCGGTA
ETHE1	GAGGACTCTGAACCCTCGGCTCACCCTCAGCTGTGAGGAGTTTGTCAAAATCATGGGCAACCTGAACTTGCCTAAACCTCAGCAGATAGACTTTGCTGTT
FCAR	TGCTGAGATTATAGGCATGAGCCACCACGCCTGGCCAGATGCATGTTCAAACCAATCAAATGGTGTTTTCTTATGCAGGACTGATCGATTTGCACCCACC
FHL2	CATTGCAACGAATCTCTCTTTGGCAAGAAGTACATCCTGCGGGAGGAGAGCCCCTACTGCGTGGTGTGCTTTGAGACCCTGTTCGCCAACACCTGCGAGG
FOS	ACTCAAGTCCTTACCTCTTCCGGAGATGTAGCAAAACGCATGGAGTGTGTATTGTTCCCAGTGACACTTCAGAGAGCTGGTAGTTAGTAGCATGTTGAGC
FOXM1	CAATTCGCCATCAACAGCACTGAGAGGAAGCGCATGACTTTGAAAGACATCTATACGTGGATTGAGGACCACTTTCCCTACTTTAAGCACATTGCCAAGC
FTH1	CCAAATACTTTCTTCACCAATCTCATGAGGAGAGGGAACATGCTGAGAAACTGATGAAGCTGCAGAACCAACGAGGTGGCCGAATCTTCCTTCAGGATAT
GADD45A	GTTACTCCCTACACTGATGCAAGGATTACAGAAACTGATGCCAAGGGGCTGAGTGAGTTCAACTACATGTTCTGGGGGCCCGGAGATAGATGACTTTGCA
GCLC	CTCAAGTGGGGCGATGAGGTGGAATACATGTTGGTATCTTTTGATCATGAAAATAAAAAAGTCCGGTTGGTCCTGTCTGGGGAGAAAGTTCTTGAAACTC
GCLM	TCATCATCAACTAGAAGTGCAGTTGACATGGCCTGTTCAGTCCTTGGAGTTGCACAGCTGGATTCTGTGATCATTGCTTCACCTCCTATTGAAGATGGAG
GGT1	CATGAATGCCCACAGCATGGGCATCGGGGGTGGCCTCTTCCTCACCATCTACAACAGCACCACACGAAAAGCTGAGGTCATCAACGCCCGCGAGGTGGCC
GLA	GCTGCTCCTTTATTCATGTCTAATGACCTCCGACACATCAGCCCTCAAGCCAAAGCTCTCCTTCAGGATAAGGACGTAATTGCCATCAATCAGGACCCCT

GNMT	GCTTGAGTAAGTTCCGGCTCTCCTACTACCCACACTGTCTGGCATCCTTCACGGAGCTGCTCCAAGCAGCCTTCGGAGGTAAGTGCCAGCACAGCGTCCT
GOT1	GTCCTCACCAACCTGGGAGAATCACAATGCTGTGTTTTCCGCTGCTGGTTTTAAAGACATTCGGTCCTATCGCTACTGGGATGCAGAGAAGAGAGGATTG
GOT2	GGAGAGTAGGAAACTGTACTTTATCTCGGCATCCTCTTGAATGATAGTGCAAGTTTCTCCAGTTGGGATGTTGTCTCTGCCCGGTTGGACCTCCTCCCTT
GPX1	GGTTTTCATCTATGAGGGTGTTTCCTCTAAACCTACGAGGGAGGAACACCTGATCTTACAGAAAATACCACCTCGAGATGGGTGCTGGTCCTGTTGATCC
GPX2	CCTCTGGTTGGTGATTCAACTTGGGCTCCAAGACTTGGGTAAGCTCTGGGCCTTCACAGAATGATGGCACCTTCCTAAACCCTCATGGGTGGTGTCTGAG
GPX3	GTCCAATTGTTCTGCTCTAACTGATACCTCAACCTTGGGGCCAGCATCTCCCACTGCCTCCAAATATTAGTAACTATGACTGACGTCCCCAGAAGTTTCT
GPX4	CAGGGAGTAACGAAGAGATCAAAGAGTTCGCCGCGGGCTACAACGTCAAATTCGATATGTTCAGCAAGATCTGCGTGAACGGGGACGACGCCCACCCGCT
GPX5	CATCCTGAAGAACATTCCTAGACATTCTGACTCTTCCATCTCTCTCTACCCTGGAGGTGTAGAAATAGCAATGGGGTCACAGTCACACAATTTAGGTTCC
GSR	ATGCAGGGACTTGGGTGTGATGAAATGCTGCAGGGTTTTGCTGTTGCAGTGAAGATGGGAGCAACGAAGGCAGACTTTGACAACACAGTCGCCATTCACC
GSS	CAACCAGGCCACGGGACCTTCTATCCTCTGTATTTGTCATTCCTCTCCTAGCCCTCCTGAGGGGTATCCTCCTAAAGACCTCCAAAGTTTTTATGGAAGG
GSTP1	TTTTGAGACCCTGCTGTCCCAGAACCAGGGAGGCAAGACCTTCATTGTGGGAGACCAGATCTCCTTCGCTGACTACAACCTGCTGGACTTGCTGCTGATC
GSTZ1	CGTAACACCACGGGGAAGGCTGTGTGCCTTTTCTCATCCGCTTTTGTTGTGTGTGACTCCAAAGAATGCCCGCGCTGAAATTTGGCGTGAATTAAACTGA
GUSB	CCGATTTCATGACTGAACAGTCACCGACGAGAGTGCTGGGGAATAAAAAGGGGATCTTCACTCGGCAGAGACAACCAAAAAGTGCAGCGTTCCTTTTGCG
HMOX1	TGTTGTTTTTATAGCAGGGTTGGGGTGGTTTTTGAGCCATGCGTGGGTGGGGAGGGAGGTGTTTAACGGCACTGTGGCCTTGGTCTAACTTTTGTGTGAA
HPRT1	TGTGATGAAGGAGATGGGAGGCCATCACATTGTAGCCCTCTGTGTGCTCAAGGGGGGCTATAAATTCTTTGCTGACCTGCTGGATTACATCAAAGCACTG
HSP90AA1	CTTGACCAATGACTGGGAAGATCACTTGGCAGTGAAGCATTTTTCAGTTGAAGGACAGTTGGAATTCAGAGCCCTTCTATTTGTCCCACGACGTGCTCCT
HSP90AB1	AGCCAATATGGAGCGGATCATGAAAGCCCAGGCACTTCGGGACAACTCCACCATGGGCTATATGATGGCCAAAAAGCACCTGGAGATCAACCCTGACCAC
HSPA1A	GCTTCCCAGAGCGAACCTGTGCGGCTGCAGGCACCGGCGCGTCGAGTTTCCGGCGTCCGGAAGGACCGAGCTCTTCTCGCGGATCCAGTGTTCCGTTTCC
HTATSF1	CTTTGATAGGCACCCAGATGGTGTGGCCTCTGTGTCCTTTCGGGATCCAGAGGAAGCTGATTATTGTATTCAGACTCTCGATGGAAGATGGTTTGGTGGC

IFNA1	ATCCCTCTCTTTATCAACAAACTTGCAAGAAAGATTAAGGAGGAAGGAATAACATCTGGTCCAACATGAAAACAATTCTTATTGACTCATACACCAGGTC
IFNB1	ACAGACTTACAGGTTACCTCCGAAACTGAAGATCTCCTAGCCTGTGCCTCTGGGACTGGACAATTGCTTCAAGCATTCTTCAACCAGCAGATGCTGTTTA
IFNG	ATACTATCCAGTTACTGCCGGTTTGAAAATATGCCTGCAATCTGAGCCAGTGCTTTAATGGCATGTCAGACAGAACTTGAATGTGTCAGGTGACCCTGAT
IL10	AAGGATCAGCTGGACAACTTGTTGTTAAAGGAGTCCTTGCTGGAGGACTTTAAGGGTTACCTGGGTTGCCAAGCCTTGTCTGAGATGATCCAGTTTTACC
IL12B	GCAAGGCTGCAAGTACATCAGTTTTATGACAATCAGGAAGAATGCAGTGTTCTGATACCAGTGCCATCATACACTTGTGATGGATGGGAACGCAAGAGAT
IL16	TCCTGTGAGACGAAGCTACTTGACGAAAAGACCAGCAAACTCTATTCTATCAGCAGCCAAGTGTCATCGGCTGTCATGAAATCCTTGCTGTGCCTTCCAT
IL1B	GGGACCAAAGGCGGCCAGGATATAACTGACTTCACCATGCAATTTGTGTCTTCCTAAAGAGAGCTGTACCCAGAGAGTCCTGTGCTGAATGTGGACTCAA
IL2	AGGATGCAACTCCTGTCTTGCATTGCACTAAGTCTTGCACTTGTCACAAACAGTGCACCTACTTCAAGTTCTACAAAGAAAACACAGCTACAACTGGAGC
IRF1	CTGTGCGAGTGTACCGGATGCTTCCACCTCTCACCAAGAACCAGAGAAAAGAAAGAAAGTCGAAGTCCAGCCGAGATGCTAAGAGCAAGGCCAAGAGGAA
IRF2	AGGCAGCACTAGCGACATTGCAGTCTGCTTCTGCACCTTATCTTAAAGCACTTACAGATAGGCCTTCTTGTGATCTTGCTCTATCTCACAGCACACTCAG
KLRD1	CAATTTTACTGGATTGGACTCTCTTACAGTGAGGAGCACACCGCCTGGTTGTGGGAGAATGGCTCTGCACTCTCCCAGTATCTATTTCCATCATTTGAAA
KRT1	TGCAGCAGGTAGATACCTCCACTAGAACCCATAATTTAGAGCCCTACTTTGAGTCATTCATCAACAATCTCCGAAGGAGAGTGGACCAACTGAAGAGTGA
LDHA	AACTTCCTGGCTCCTTCACTGAACATGCCTAGTCCAACATTTTTTCCCAGTGAGTCACATCCTGGGATCCAGTGTATAAATCCAATATCATGTCTTGTGC
LHPP	ACCATACCTGCTCATCCATGACGGAGTCCGCTCAGAATTTGATCAGATCGACACATCCAACCCAAACTGTGTGGTAATTGCAGACGCAGGAGAAAGCTTT
LPO	CTGCTGGCCAAGAAATCCAAGCTGATGAAACAGAATAAAATGATGACTGGAGAGCTGCGCAACAAGCTTTTCCAGCCAACTCACAGGATCCATGGCTTTG
LTBR	GTGCTGCCTGGGCCCTCGAGTGTACACACTGCGAGCTACTTTCTGACTGCCCGCCTGGCACTGAAGCCGAGCTCAAAGATGAAGTTGGGAAGGGTAACAA
MAP3K5	TTTAGGAAAAGGCACTTATGGGATAGTCTACGCAGGTCGGGACTTGAGCAACCAAGTCAGAATTGCTATTAAGGAAATCCCAGAGAGAGACAGCAGATAC
MAT1A	GGGGATTAACCCACTAGCTCTTGTTGAGCCGTGGGCAATTGTCTGAAAAGTGAAGACAGAACCACAGGGCTATTTTGTTTGCTTCATGTGTCCCAGAAGA
MAT2A	AAGCAGTTGTGCCTGCGAAATACCTTGATGAGGATACAATCTACCACCTACAGCCAAGTGGCAGATTTGTTATTGGTGGGCCTCAGGGTGATGCTGGTTT

MB	CAAGAGAGAGCGGGGTCTGATCTCGTGTAGCCATATAGAGTTTGCTTCTGAGTGTCTGCTTTGTTTAGTAGAGGTGGGCAGGAGGAGCTGAGGGGCTGGG
MBL2	TGGACTTGTCTTTTGGTGGACATGGTGCACTAATTTCACTACCTATCCAGGAGTGGAACTGGTAGAGGATGAGGAAAGCATGTATTCAGCTTTAGTAGAT
MPO	AGACCTGCTGGAGAGGAAGCTGCGGTCCCTGTGGCGAAGGCCATTCAATGTCACTGATGTGCTGACGCCCGCCCAGCTGAATGTGTTGTCCAAGTCAAGC
MPST	CGCGACGCGCGACGCGAGTTCGAGGAGCGCCACATCCCGGGCGCCGCTTTCTTCGACATCGACCAGTGCAGCGACCGCACCTCGCCCTACGACCACATGC
MPV17	TGGTACAAGGTTTTGGATCGGTTCATCCCTGGCACCACCAAAGTGGATGCACTGAAGAAGATGTTGTTGGATCAGGGGGGCTTTGCCCCGTGTTTTCTAG
MSRA	TGCAGAAGTCGTCCGAGTGGTGTACCAGCCAGAACACATGAGTTTTGAGGAACTGCTCAAGGTCTTCTGGGAGAATCACGACCCGACCCAAGGTATGCGC
MT3	CACTAGCCAAGCCGCGCGTCCAGTTGCTTGGAGAAGCCCGTTCACCGCCTCCAGCTGCTGCTCTCCTCGACATGGACCCTGAGACCTGCCCCTGCCCTTC
MTR	TGAGTGAACTACAGCTATACCTCACAATAAGAATGAATCTCAGAAAATATTAAGGAAAAAAGCAAGTTTGAAGAGACCACATGGGGCGTACTATTTTTAT
NCF1	TGAGCCTGCCCACCAAGATCTCCCGCTGTCCCCACCTCCTCGACTTCTTCAAGGTGCGCCCTGATGACCTCAAGCTCCCCACGGACAACCAGACAAAAAA
NCF2	GCTCACCGTGTGCTATTTGGGTTTGTGCCTGAGACAAAAGAAGAGCTCCAGGTCATGCCAGGGAACATTGTCTTTGTCTTGAAGAAGGGCAATGATAACT
NCOA7	TCAGGATCTGGAGGTGTGGGCATTTGATTGAAATTCAGACTGCCTTAAAATATAACATTAAAAAGACTGGGTTCGATCAGCCCTCCTAAAGCTGGCTGGA
NFATC1	CGAATTCTCTGGTGGTTGAGATCCCGCCATTTCGGAATCAGAGGATAACCAGCCCCGTTCACGTCAGTTTCTACGTCTGCAACGGGAAGAGAAAGCGAAG
NFKBIA	GGATGAGGAGAGCTATGACACAGAGTCAGAGTTCACGGAGTTCACAGAGGACGAGCTGCCCTATGATGACTGTGTGTTTGGAGGCCAGCGTCTGACGTTA
NOS2	CCCCAGCGGAGTGATGGCAAGCACGACTTCCGGGTGTGGAATGCTCAGCTCATCCGCTATGCTGGCTACCAGATGCCAGATGGCAGCATCAGAGGGGACC
NOX4	ATAGCAAAATATAACAGAGGAAAAACAGTTGGTGTTTTCTGTTGTGGACCCAATTCACTATCCAAGACTCTTCATAAACTGAGTAACCAGAACAACTCAT
NOX5	AGCTCCATAAGGTGGACTTTATCTGGATCAACAGAGACCAGCGGTCTTTCGAGTGGTTTGTGAGCCTGCTGACTAAACTGGAGATGGACCAGGCCGAGGA
NQO1	CCGAATTCAAATCCTGGAAGGATGGAAGAAACGCCTGGAGAATATTTGGGATGAGACACCACTGTATTTTGCTCCAAGCAGCCTCTTTGACCTAAACTTC
NUDT1	TGTGGCCCGACGACAGCTACTGGTTTCCACTCCTGCTTCAGAAGAAGAAATTCCACGGGTACTTCAAGTTCCAGGGTCAGGACACCATCCTGGACTACAC
PDLIM1	GGATCCCAACAAGCCCTCAGGATTCAGAAGTGTTAAAGCTCCTGTCACTAAAGTGGCTGCGTCGATTGGAAATGCTCAGAAGTTGCCTATGTGTGACAAA

POLR1B	GGAGAACTCGGCCTTAGAATACTTTGGTGAGATGTTAAAGGCTGCTGGCTACAATTTCTATGGCACCGAGAGGTTATATAGTGGCATCAGTGGGCTAGAA
PPIA	GGAATATTGAAAATGTAGGCAGCAACTGGGCATGGTGGCTCACTGTCTGTAATGTATTACCTGAGGCAGAAGACCACCT
PRDX1	GACCCATGAACATTCCTTTGGTATCAGACCCGAAGCGCACCATTGCTCAGGATTATGGGGTCTTAAAGGCTGATGAAGGCATCTCGTTCAGGGGCCTTTT
PRDX2	GTCGGACTACAAAGGGAAGTACGTGGTCCTCTTTTTCTACCCTCTGGACTTCACTTTTGTGTGCCCCACCGAGATCATCGCGTTCAGCAACCGTGCAGAG
PRDX3	CATGGTTAGTTGCTAGTACAAGGAATCCTTTATTGGTAACATCTTGGTGGCTGGCTAGCTAGTTTCTACAGAACATAATTTGCCTCTATAGAAGGCTATT
PRDX4	AGGAGGACTTGGGCCAATAAGGATTCCACTTCTTTCAGATTTGACCCATCAGATCTCAAAGGACTATGGTGTATACCTAGAGGACTCAGGCCACACTCTT
PRDX5	GGAAGGAGACAGACTTATTACTAGATGATTCGCTGGTGTCCATCTTTGGGAATCGACGTCTCAAGAGGTTCTCCATGGTGGTACAGGATGGCATAGTGAA
PRDX6	GTGATAAGTTTCTATCAAAATGGGGAGATTGCAGAAAAGGCTTCCCTTGGCTCCCAAGGAGGTGTAGCAGGTGTGAGCAATATTAGTGCCATGTGCCTTT
PRNP	GCATGAGCTCTGTGTGTACCGAGAACTGGGGTGATGTTTTACTTTTCACAGTATGGGCTACACAGCAGCTGTTCAACAAGAGTAAATATTGTCACAACAC
PTGR1	TGATAATGTAGGTGGAGAGTTTTCAAACACTGTTATCGGCCAGATGAAGAAATTTGGAAGGATTGCCATATGTGGAGCCATCTCTACATATAACAGAACC
PTGS1	ACCCCCAAGGCACCAACCTCATGTTTGCCTTCTTTGCACAACACTTCACCCACCAGTTCTTCAAAACTTCTGGCAAGATGGGTCCTGGCTTCACCAAGGC
PTGS2	GCTACAAAAGCTGGGAAGCCTTCTCTAACCTCTCCTATTATACTAGAGCCCTTCCTCCTGTGCCTGATGATTGCCCGACTCCCTTGGGTGTCAAAGGTAA
PTK2B	CCAGTAGATGTGGAAAAGGAGGACGTGCGTATCCTCAAGGTCTGCTTCTATAGCAACAGCTTCAATCCTGGGAAAAACTTCAAACTGGTCAAATGCACTG
RBL2	GAATCTCCACACCACTAACTGGTGTTAGGTACATTAAGGAGAATAGCCCTTGTGTGACTCCAGTTTCTACAGCTACGCATAGCTTGAGTCGTCTTCACAC
RNF7	TTCAGAGCCCTGGTGGATCTTGTAATCCAGTGCCCTACAAAGGCTAGAACACTACAGGGGATGAATTCTTCAAATAGGAGCCGATGGATCTGTGGTCCTT
RPLP0	CGAAATGTTTCATTGTGGGAGCAGACAATGTGGGCTCCAAGCAGATGCAGCAGATCCGCATGTCCCTTCGCGGGAAGGCTGTGGTGCTGATGGGCAAGAA
SEPP1	GGAGCATAAGAGATCAAGATCCAATGCTAAACTCCAATGGTTCAGTGACTGTGGTTGCTCTTCTTCAAGCCAGCTGATACCTGTGCATACTGCAGGCATC
VIMP	GAAATGCAAAGAAGCCCCAGGAGGAAGACAGTCCTGGGCCTTCCACTTCATCTGTCCTGAAACGGAAATCGGACAGAAAGCCTTTGCGGGGAGGAGGTTA
SELL	CTAACTCCAGTGAAGTAATGGGGTCCTGCTCAAGTTGAAAGAGTCCTATTTGCACTGTAGCCTCGCCGTCTGTGAATTGGACCATCCTATTTAACTGGCT

SERPINA1	TCACTGTCAACTTCGGGGACACCGAAGAGGCCAAGAAACAGATCAACGATTACGTGGAGAAGGGTACTCAAGGGAAAATTGTGGATTTGGTCAAGGAGCT
SERPINC1	TCAAGGCCAACAGGCCTTTCCTGGTTTTTATAAGAGAAGTTCCTCTGAACACTATTATCTTCATGGGCAGAGTAGCCAACCCTTGTGTTAAGTAAAATGT
SFTPD	AAAGTGGGCTTCCAGATGTTGCTTCTCTGAGGCAGCAGGTTGAGGCCTTACAGGGACAAGTACAGCACCTCCAGGCTGCTTTCTCTCAGTATAAGAAAGT
SIRT2	CTATGACAACCTAGAGAAGTACCATCTTCCCTACCCAGAGGCCATCTTTGAGATCAGCTATTTCAAGAAACATCCGGAACCCTTCTTCGCCCTCGCCAAG
SLC7A11	TCCATTACCAGCTTTTGTACGAGTCTGGGTGGAACTCCTCATAATACGCCCTGCAGCTACTGCTGTGATATCCCTGGCATTTGGACGCTACATTCTGGAA
SLPI	TTTCTGTGAGATGGATGGCCAGTGCAAGCGTGACTTGAAGTGTTGCATGGGCATGTGTGGGAAATCCTGCGTTTCCCCTGTGAAAGCTTGATTCCTGCCA
SMARCB1	AGAAGGAGAACTCACCAGAGAAGTTTGCCCTGAAGCTGTGCTCGGAGCTGGGGTTGGGCGGGGAGTTTGTCACCACCATCGCATACAGCATCCGGGGACA
SOD1	GCCTATAAAGTAGTCGCGGAGACGGGGTGCTGGTTTGCGTCGTAGTCTCCTGCAGCGTCTGGGGTTTCCGTTGCAGTCCTCGGAACCAGGACCTCGGCGT
SOD2	TTTGGGGTATCTGGGCTCCAGGCAGAAGCACAGCCTCCCCGACCTGCCCTACGACTACGGCGCCCTGGAACCTCACATCAACGCGCAGATCATGCAGCTG
SOD3	GGTGCAGCTCTCTTTTCAGGAGAGAAAGCTCTCTTGGAGGAGCTGGAAAGGTGCCCGACTCCAGCCATGCTGGCGCTACTGTGTTCCTGCCTGCTCCTGG
SPINK1	GACGTGGTAAGTGCGGTGCAGTTTTCAACTGACCTCTGGACGCAGAACTTCAGCCATGAAGGTAACAGGCATCTTTCTTCTCAGTGCCTTGGCCCTGTTG
SQRDL	AAAGAGTTCCTTGATGGGTAATGGTGACCAAATGCCTCCCTTTTCAGTACCTTTGAACAGCAACCATGTGGGCTACTCATGATGGGCTTGATTCTTTGGG
SQSTM1	GGGCCAGTTTCTCTGCCTTCTTCCAGGATCAGGGGTTAGGGTGCAAGAAGCCATTTAGGGCAGCAAAACAAGTGACATGAAGGGAGGGTCCCTGTGTGTG
SRXN1	AAGAGAGTGAGAGTAGAAGCTGAAAGACTTCTTGAGTTCTTGGCCTGGAACTGGGACTAGGACAGTGTCACTTCTGCTAAGTTCTTTTGGTCAGAGCAAA
STAT1	ACAGTGGTTAGAAAAGCAAGACTGGGAGCACGCTGCCAATGATGTTTCATTTGCCACCATCCGTTTTCATGACCTCCTGTCACAGCTGGATGATCAATAT
STAT3	AAAGAAGGAGGCGTCACTTTCACTTGGGTGGAGAAGGACATCAGCGGTAAGACCCAGATCCAGTCCGTGGAACCATACACAAAGCAGCAGCTGAACAACA
SUOX	GGCGGGATTACAAAGGCTTCTCTCCATCTGTGGACTGGGAGACTGTAGATTTTGACTCTGCTCCATCCATTCAGGAACTTCCTGTCCAGTCGGCCATCAC
TGFB1	TATATGTTCTTCAACACATCAGAGCTCCGAGAAGCGGTACCTGAACCCGTGTTGCTCTCCCGGGCAGAGCTGCGTCTGCTGAGGCTCAAGTTAAAAGTGG
TNF	AGCAACAAGACCACCACTTCGAAACCTGGGATTCAGGAATGTGTGGCCTGCACAGTGAAGTGCTGGCAACCACTAAGAATTCAAACTGGGGCCTCCAGAA

TNFRSF1B	CCCAGCTGAAGGGAGCACTGGCGACTTCGCTCTTCCAGTTGGACTGATTGTGGGTGTGACAGCCTTGGGTCTACTAATAATAGGAGTGGTGAACTGTGTC
TNFSF10	GGGGGGACCCAGCCTGGGACAGACCTGCGTGCTGATCGTGATCTTCACAGTGCTCCTGCAGTCTCTCTGTGTGGCTGTAACTTACGTGTACTTTACCAAC
TPO	CTCCAGCTCAGCTTCTGTCTTTTTCCAAACTTCCTGAGCCAACAAGCGGAGTGATTGCCCGAGCAGCAGAGATAATGGAAACATCAATACAAGCGATGAA
TRAPPC6A	CCTGCGCGGCGCCCTCTATACCCTGGGCATTGAGAGCGTGGTCACCGCCTCCGTGGCAGCCCTGCCCGTCTGTAAGTTCCAGGTGGTGATTCCGAAATCC
TRIM5	GAAAGCTTCCTGGAAGACTCAAATACAGTATGACAAAACCAACGTCTTGGCAGATTTTGAGCAACTGAGAGACATCCTGGACTGGGAGGAGAGCAATGAG
TST	CCTCTTAGGTGAAGGAGATGACATGTTTTTAGAATTGCTGTGCAAGGCTCACCCTCTCTCTGTCAACACTGGAATAAACTTTGCCTTTTCTGAGTAGTGA
TTN	GTGCCTGAGGCTCCCAAAGAAGTTGTTCCTGAAAAGAAAGTGCCAGTGCCTCCTCCTAAAAAGCCTGAAGTGCCACCCACAAAAGTCCCAGAGGTGCCAA
TXN	CAGCCAAGATGGTGAAGCAGATCGAGAGCAAGACTGCTTTTCAGGAAGCCTTGGACGCTGCAGGTGATAAACTTGTAGTAGTTGACTTCTCAGCCACGTG
TXNRD1	AGTGATGATCTTTTCTCCTTGCCTTACTGCCCGGGTAAGACCCTGGTTGTTGGAGCATCCTATGTCGCTTTGGAGTGCGCTGGATTTCTTGCTGGTATTG
TXNRD2	TTTAACATCAAAGCCAGCTTTGTTGACGAGCACACGGTTTGCGGCGTTGCCAAAGGTGGGAAAGAGATTCTGCTGTCAGCCGATCACATCATCATTGCTA
UCP2	CTGCCGTGTTCTCCCTGCGGCTCGGACACATAGTATGACCATTAGGTGTTTCGTCTCCCACCCATTTTCTATGGAAAACCAAGGGGATCGGGCCATGATA
VPS4A	CAAGCTCTGCCTCAAAGACCGAGTGACATAAGCCATTCCCACCCTCCTAGGTTCACATCCAGGGCTGTGTCTTCCTTGGGGGAGGAGATGGTGTCGTTTA
XCL1	CCTCACTACCCAGCGACTGCCGGTTAGCAGAATCAAGACCTACACCATCACGGAAGGCTCCTTGAGAGCAGTAATTTTTATTACCAAACGTGGCCTAAAA
XPO1	AATCTTTCTTCAGGAATATGTGGCTAATCTCCTTAAGTCGGCCTTCCCTCACCTACAAGATGCTCAAGTAAAGCTCTTTGTGACAGGGCTTTTCAGCTTA
YY1	GACCCTGGAGGGCGAGTTCTCGGTCACCATGTGGTCCTCAGATGAAAAAAAAGATATTGACCATGAGACAGTGGTTGAAGAACAGATCATTGGAGAGAAC

Appendix Table S2. Normalized intensity values of differentially expressed gene in nCounter Gene Expression Assay

Probe Name	Accession No.	[a]UT1	UT2	UT3	[b]PMA1	PMA2	PMA3	[c]Vs1	Vs2	Vs3	[d]Vs+PMA1	Vs+PMA2	Vs+PMA3
APOBEC3G	NM_021822.2	117.74	93.09	111.61	87.34	113.45	101.48	107.99	97.01	130.42	61.14	44.6	72.24
BAD	NM_004322.3	225.19	187.96	150.62	85.15	146.97	104.26	25.6	26.95	42.87	22.48	37.66	35.14
BAG2	NM_004282.3	835.59	732.16	1116.11	424.67	523.4	760.43	422.35	411.14	540.83	210.39	174.43	274.32
BANF1	NM_001143985.1	2553.64	2298.53	2576.81	1136.45	1170.57	1433.27	1215.85	1169.51	1543.14	811.9	668.97	1234.92
BAX	NM_138761.3	3456.68	3294.74	3130.53	2042.55	2029.16	1864.22	1414.23	1331.97	1765.68	636.57	546.08	1118.75
BCL2	NM_000657.2	587.54	564.79	494.12	164.84	167.59	197.4	137.58	157.83	228.92	119.58	78.29	96.65
BNIP3	NM_004052.2	863.03	750.96	1115.03	364.62	453.79	486.56	639.12	436.55	536.27	738.17	969.27	257.72
CASP3	NM_032991.2	1707.76	1654.98	1758.69	1118.98	1175.73	1498.61	959.88	947.78	1072.54	724.68	506.44	774.14
CASP8	NM_001228.4	948.76	826.15	786.7	852.61	912.73	804.91	675.92	682.92	606.5	439.67	232.9	391.46
CAT	NM_001752.2	10451.2	9596.01	10668.1	1033.83	1309.8	1832.25	2229.33	2223.54	3752.98	2005.02	1654.1	3383.57
CCL2	NM_002982.3	124.6	103.83	71.52	2105.87	1926.02	955.05	7369.52	7910.96	5013.39	4399.36	2011.88	1972.94
CCL3	NM_002983.2	145.17	104.72	136.53	57120.4	45701.2	60588	2458.9	2428.34	1879.68	16935.7	19038.5	11215.8
CCL4	NM_002984.2	17.48	15.07	15.19	8804.47	10236	9745.12	181.58	207.88	116.74	1960.96	6464.77	3311.33
CCL8	NM_005623.2	17.48	15.07	15.19	16.82	22.63	15.02	173.58	185.55	46.51	22.48	30.72	16.32
CCR2	NM_001123041.2	25.15	25.96	28.17	19.65	61.88	15.02	17.91	18.48	14.69	16.09	11.62	20.5
CDK7	NM_001799.2	632.12	635.5	659.92	569.86	538.87	703.43	255.97	227.9	347.48	232.87	246.78	320.2
CDK9	NM_001261.2	608.12	634.6	599.23	425.76	355.81	346.15	277.57	255.61	384.87	208.59	228.94	165.96
CDKN1A	NM_000389.2	352.07	350.87	248.15	7788.11	7044.04	11920.7	1926.17	1966.38	1427.32	3260.18	2727.43	2692.41
CEBPB	NM_005194.2	6587.58	5936.08	4550.06	11952.9	10344.3	9907.77	5682.52	5585.8	5743.01	6426.86	4996	4635.09
COPS6	NM_006833.4	9499.01	8990.05	9159.71	4711.73	4496.63	4826.68	3989.12	3825.75	5850.63	3092.05	2338.93	4694.64
CXCL8	NM_000584.2	1827.79	1543.99	2689.51	61420.6	58154.6	156598	51975.4	45290.8	97424.2	90665.6	235659	161405
CXCR4	NM_003467.2	20556	20851.5	17061.4	475.98	502.78	247.45	3118.83	2744.78	2085.8	1186.83	3079.26	185.48
DHCR24	NM_014762.2	2024.4	3779.87	1095.52	2049.1	2178.7	2047.73	629.52	654.44	778.87	682.43	386.52	438.32
FOS	NM_005252.2	516.67	480.65	595.98	1429.02	1327.85	1391.56	432.75	422.69	476.99	684.22	1427.15	615.02
FOXM1	NM_202002.1	2035.83	2042.54	1984.08	1043.65	1147.36	1402.69	1256.65	1319.65	1629.79	1060.05	600.59	1165.6
FTH1	NM_002032.2	62320.8	76603.4	54991.9	319563	306692	298112	110951	112593	119137	156265	153698	149725
GCLC	NM_001498.2	1377.41	1209.23	1322	1227.06	1206.67	1577.85	489.54	454.25	662.13	355.15	246.78	381.7

GGT1	NM_001032364.2	189.75	200.49	173.38	58.95	105.71	73.68	155.98	148.6	176.93	180.72	140.73	132.77
GPX1	NM_000581.2	3066.89	1891.28	2350.34	900.64	1039.07	924.47	788.71	927.76	1016.91	438.77	320.12	620.88
GPX4	NM_001039847.1	7666.65	7403.1	6995.75	6132.02	7361.17	6732.61	2885.25	3113.57	4016.55	3431.91	3125.85	3985.9
GSS	NM_000178.2	941.9	847.63	892.89	418.12	520.83	560.24	259.17	267.16	398.55	224.78	180.38	335.82
GSTP1	NM_000852.2	24463.1	21326.8	24477.5	8404.91	8518.85	12620	10428.4	10586.5	14563.2	9960.37	8049.5	12209.6
HSP90AA1	NM_001017963.2	38620.2	37539.1	36804.6	19307.6	22001	26589.9	19052.9	19369.7	23375.2	14271.6	10324	19865.1
IL10	NM_000572.2	17.48	17.01	15.19	16.82	22.63	41.71	167.98	131.66	51.07	16.09	29.73	20.5
IL16	NM_001172128.1	83.44	98.46	69.35	80.78	108.29	65.34	36.8	40.04	57.46	39.56	29.73	27.33
IL1B	NM_000576.2	21.72	23.27	15.19	5568.7	4576.56	4611.21	1302.24	1325.81	1656.23	3205.34	19506.3	3593.46
IRF1	NM_002198.1	86.87	98.46	70.43	70.96	134.07	102.87	318.36	332.61	294.58	332.67	332.01	420.75
LTBR	XM_006718983.2	957.9	877.17	825.71	591.7	685.84	614.46	307.16	294.88	369.37	213.99	163.53	178.65
MAT2A	NM_005911.4	9373.27	7044.17	5776.7	4114.57	3797.9	3283.59	4259.49	4057.5	5530.51	3521.82	2729.42	3371.86
NCF1	NM_000265.4	2736.54	2482.02	1958.07	10164.7	9060.3	4669.59	736.71	724.5	585.52	300.3	296.33	239.17
NCF2	NM_000433.3	549.82	486.92	507.13	2494.51	2506.15	1544.48	963.08	990.89	848.18	311.99	219.03	264.56
NCOA7	NM_181782.3	315.49	277.47	249.23	215.06	221.74	241.89	352.76	390.35	672.16	469.34	575.81	949.86
NFKBIA	NM_020529.1	1314.54	1219.98	1208.22	3148.43	3065.65	5334.1	5361.76	5352.51	6410.61	3623.42	10234.8	7776.56
NOS2	NM_000625.4	32.01	15.22	20.59	52.4	139.23	52.83	32	16.17	20.98	25.18	25.77	30.26
NQO1	NM_000903.2	2116.99	1921.71	2040.43	2295.82	2511.31	2560.7	1275.85	1320.42	1804.89	800.21	686.81	1016.24
NUDT1	NM_002452.3	1114.5	1049.91	1035.93	421.39	381.59	546.34	411.15	417.3	655.74	329.08	282.46	522.28
PRDX6	NM_004905.2	2902.28	2828.41	2992.92	1991.24	1975.01	2247.91	1017.48	967.79	1700.01	918.89	764.12	1490.69
PTGS1	NM_000962.2	853.88	842.26	765.02	741.26	868.9	617.24	268.77	253.3	358.43	412.69	227.95	365.11
PTGS2	NM_000963.1	25.15	27.75	27.09	269.65	275.88	280.82	183.18	180.93	142.28	225.68	1292.36	267.48
RBL2	NM_005611.3	1012.77	922.81	733.6	462.88	466.68	360.05	221.57	247.92	296.41	171.73	111.99	197.2
SIRT2	NM_012237.3	1890.66	1909.18	1327.42	1135.36	1191.2	911.95	684.72	675.22	686.75	566.44	435.08	588.66
SLC7A11	NM_014331.3	597.83	688.31	1928.82	6604.72	6556.73	9800.73	4368.28	4349.3	7196.78	5595.18	5264.58	7098.09
SMARCB1	NM_003073.3	1444.86	1207.44	1375.09	818.77	737.41	975.9	414.35	438.09	445.98	259.84	170.46	370.96
SOD1	NM_000454.4	294.92	257.78	263.32	234.71	278.46	275.25	130.38	134.74	134.07	113.29	112.98	154.24
SOD2	NM_000636.2	1665.47	1585.16	1815.04	1568.76	1513.49	1441.61	5300.16	5273.21	5572.46	3248.49	24471.6	5511.73
SQRDL	NM_021199.2	7146.55	7134.58	9256.15	8510.8	8245.55	9896.65	4395.47	4069.82	5632.66	3488.56	2417.23	4607.75
SQSTM1	NM_003900.3	7894.12	8820.88	8751.19	18880.8	22168.6	24981.4	10975.5	11073	12606	9527.89	9885.96	10681.8
SRXN1	NM_080725.1	841.31	901.33	1664.42	2573.11	2810.4	6443.46	1870.18	1833.19	4130.56	1337.88	2206.13	4374.44

STAT3	NM_003150.3	887.03	912.97	756.36	906.1	935.94	779.89	1086.27	1160.27	1091.69	1670.55	1264.61	1513.14
TGFB1	NM_000660.3	4524.32	3944.56	3916.15	9002.07	7765.97	8303.51	1766.99	1874.76	2495.3	1864.76	1182.35	2052.99
TNF	NM_000594.2	563.54	486.92	545.05	4455.18	4225.91	4803.05	882.29	860.77	1349.8	3558.69	17077.2	11785.9
TNFRSF1B	NM_001066.2	868.74	807.35	702.18	2259.8	2212.22	2076.92	929.49	990.89	851.83	770.54	875.12	466.63
TNFSF10	NM_003810.2	118.88	81.45	91.02	26.2	22.63	15.02	63.99	56.97	32.83	28.77	31.71	28.31
TXNRD2	NM_006440.3	812.73	732.16	631.74	448.68	440.9	443.47	315.96	351.09	363.9	251.75	185.33	275.29
VPS4A	NM_013245.2	1071.07	983.68	967.66	903.92	825.07	1039.85	370.36	383.42	389.43	351.55	260.65	390.49
XCL1	NM_002995.2	17.48	15.07	15.19	16.82	22.63	27.8	24.8	19.25	29.18	18.88	20.81	31.24
XPO1	NM_003400.3	12952.3	11184.8	12082.2	6666.94	6270.53	7894.8	6265.65	5987.7	6107.82	3983.97	2300.28	3629.58
YY1	NM_003403.3	3752.74	3392.3	3611.65	1931.2	1879.61	2270.15	2203.74	2317.47	2713.27	2228.9	1683.83	2276.54

[a] UT- RNA isolated from untreated U1 cells

[b] PMA - RNA isolated from U1 cells treated with PMA (5 ng/mL)

[c] Vs - RNA isolated from U1 cells treated with Vs (50 ng/µL)

[d] Vs + PMA - RNA isolated from Vs pre-treated U1 cells exposed to PMA

Appendix Table S3. Fold change (FC>1.5, p<0.05) of differentially expressed gene in nCounter Gene Expression Assay

Probe Name	PMA v/s UT	P value
CCL4	511.59	0.00000008
CCL3	423.95	0.00000174
IL1B	235.02	0.00000109
CXCL8	41.93	0.00198838
CDKN1A	27.73	0.00014352
CCL2	16.12	0.00146677
PTGS2	10.34	0.00002098
TNF	8.46	0.00000429
SLC7A11	8.12	0.01997531
FTH1	4.81	0.00272844
NCF2	4.14	0.00977636
SRXN1	3.33	0.03362251
NOS2	3.2	0.04910377
NCF1	3.19	0.02760218
NFKBIA	2.98	0.02410768
TNFRSF1B	2.76	0.00123007
FOS	2.61	0.00196774
SQSTM1	2.58	0.00255652
TGFB1	2.03	0.00039086
CEBPB	1.9	0.0139956
SQRDL	1.14	0.29039544
SIRT2	-1.57	0.04193898
SMARCB1	-1.6	0.01250738
TXNRD2	-1.62	0.0215102
CDK9	-1.64	0.0123567
BAX	-1.66	0.00024262
HSP90AA1	-1.68	0.02845565

FOXM1	-1.7	0.02488089
XPO1	-1.74	0.00449176
YY1	-1.77	0.00345957
GSS	-1.8	0.01390084
MAT2A	-1.95	0.02567225
COPS6	-1.97	0.00002229
BANF1	-1.99	0.00369122
RBL2	-2.07	0.00500632
BNIP3	-2.08	0.00877994
NUDT1	-2.4	0.01187469
GSTP1	-2.42	0.01431701
GGT1	-2.43	0.0288025
GPX1	-2.51	0.0154972
BCL2	-3.11	0.00013608
TNFSF10	-4.14	0.00140119
CAT	-7.56	0.00525151
CXCR4	-49.8	0.00185865
**Probe Name**	**Vs v/s UT**	**P value**
IL1B	74.88	0.00030003
CCL2	68.11	0.00004715
CXCL8	31.15	0.00052114
CCL3	17.55	0.00003172
CCL4	11.64	0.00300272
CCL8	8.13	0.04187619
IL10	6.83	0.03037311
PTGS2	6.3	0.00068869
CDKN1A	5.61	0.00040328
SLC7A11	5.56	0.02786311
NFKBIA	4.56	0.0003011

IRF1	3.73	0.00240662
SOD2	3.19	0.00020199
TNF	1.9	0.03868206
NCF2	1.81	0.00082957
FTH1	1.78	0.02239238
XCL1	1.71	0.03372431
CCR2	-1.66	0.0175419
SQRDL	-1.67	0.01752221
BNIP3	-1.69	0.03047047
CASP3	-1.72	0.00158106
HSP90AA1	-1.84	0.00904793
IL16	-1.89	0.02332919
BANF1	-1.9	0.00894783
BAG2	-1.94	0.01545288
XPO1	-1.97	0.00197068
GSTP1	-2	0.01345947
CDK9	-2.04	0.02754754
SOD1	-2.04	0.00217802
TGFB1	-2.04	0.01129445
COPS6	-2.06	0.03164966
TXNRD2	-2.1	0.00236314
BAX	-2.2	0.00670195
NUDT1	-2.21	0.03307201
GPX4	-2.22	0.01107939
CDK7	-2.36	0.01988693
PRDX6	-2.45	0.03717153
GCLC	-2.46	0.00988473
SIRT2	-2.47	0.01687004
VPS4A	-2.64	0.00014599
GPX1	-2.64	0.00837197
LTBR	-2.75	0.00065299

PTGS1	-2.83	0.00616677
GSS	-2.96	0.01283299
SMARCB1	-3.09	0.0005954
BCL2	-3.21	0.01012036
RBL2	-3.48	0.0006676
NCF1	-3.49	0.00080251
CAT	-3.86	0.01382443
BAD	-6	0.00139364
CXCR4	-7.43	0.00056538
**Probe Name**	**Vs+PMA v/s UT**	**P value**
IL1B	314.28	0.00708343
CCL4	232.23	0.00345796
CCL3	120.33	0.00006001
CXCL8	76.88	0.00058601
CCL2	26.63	0.00111755
TNF	16.86	0.02608006
PTGS2	16.04	0.03748462
CDKN1A	9.21	0.00041176
SLC7A11	6.41	0.03120074
NFKBIA	5.3	0.03218853
IRF1	4.26	0.00041031
FTH1	2.39	0.01104969
NCOA7	2.27	0.04803209
STAT3	1.73	0.0070805
YY1	-1.75	0.02127443
APOBEC3G	-1.84	0.0319583
NCF2	-1.96	0.01438607
GPX4	-2.1	0.00426313

SOD1	-2.16	0.00923678
MAT2A	-2.27	0.01297646
SQRDL	-2.3	0.03057552
GSTP1	-2.35	0.01105065
CDK7	-2.43	0.01077821
NQO1	-2.46	0.01188985
CASP8	-2.49	0.03473384
TGFB1	-2.49	0.02615612
PTGS1	-2.52	0.03238737
CASP3	-2.6	0.01717971
IL16	-2.61	0.00325617
HSP90AA1	-2.63	0.03544743
BANF1	-2.83	0.02518634
COPS6	-2.85	0.03477488
PRDX6	-2.86	0.0333126
NUDT1	-2.92	0.02694722
VPS4A	-3.05	0.0081988
CDK9	-3.08	0.005854
TXNRD2	-3.08	0.00281402
SIRT2	-3.21	0.00192783
TNFSF10	-3.24	0.00521762
GSS	-3.74	0.01637243
XPO1	-3.75	0.01218524
GCLC	-4.04	0.0059256
BAG2	-4.08	0.00149844
DHCR24	-4.17	0.039298
BAX	-4.51	0.0189018
CAT	-4.57	0.01743295
LTBR	-4.81	0.00034532
SMARCB1	-5.27	0.01395405
GPX1	-5.39	0.00287154

RBL2	-5.65	0.00248872
BCL2	-5.66	0.00159778
BAD	-5.98	0.00131385
NCF1	-8.55	0.0001136
**Probe Name**	**Vs+PMA v/s PMA**	**P value**
IRF1	3.62	0.01044566
NCOA7	2.81	0.03551667
GGT1	1.94	0.03935274
STAT3	1.69	0.00840496
APOBEC3G	-1.72	0.0415904
BCL2	-1.82	0.02385733
CDK9	-1.88	0.00788836
TXNRD2	-1.9	0.03305617
CASP3	-1.91	0.01986873
GPX4	-1.92	0.00240168
FTH1	-2.01	0.00003832
CEBPB	-2.02	0.00716749
SIRT2	-2.04	0.00504856
GSS	-2.08	0.03870124
SOD1	-2.09	0.00769812
XPO1	-2.15	0.03193456
SQSTM1	-2.18	0.0046803
PTGS1	-2.26	0.02714952
CDK7	-2.27	0.00333011
CASP8	-2.5	0.03865341
BAG2	-2.56	0.01373747
IL16	-2.61	0.00778681
SQRDL	-2.61	0.02750844
BAX	-2.71	0.04251708

RBL2	-2.74	0.01386341
VPS4A	-2.79	0.0044181
NQO1	-2.98	0.00686467
CDKN1A	-3.01	0.01214941
TNFRSF1B	-3.21	0.02442046
SMARCB1	-3.3	0.02314604
LTBR	-3.42	0.00068482
CCL3	-3.52	0.00581069
BAD	-3.53	0.00515393
GCLC	-4.12	0.00182672
DHCR24	-4.29	0.01277996
TGFB1	-5.04	0.00783462
NCF2	-8.11	0.0009037
NCF1	-27.24	0.00293334

Appendix Table S4. Details of HIV-infected subject used in the study.

SL. NO.	Subject code	Duration of infection	$CD4^+$ T cell count (cell/mm3)[#]	Viral load (copies/ml)[$]
1	Subject A	9 years	327	Target not detected
2	Subject B	6 years	642	Target not detected
3	Subject C	11 years	351	Target not detected

[#] $CD4^+$ T cell count was evaluated by flow cytometry.

[$] Viral copies were evaluated by p24 ELISA. Detection limit – 20 pg/mL.

Appendix Table S5. List of primers used in the study.

Primer sequences	
Total HIV *gag* and β-actin mRNA in cell lines	
HIV *gag* F	ATAATCCACCTATCCCAGTAGGAGAAAT
HIV *gag* R	TTGGTTCCTTGTCTTATGTCCAGAATGC
β-actin F	ATGTGGCCGAGGACTTTGATT
β-actin R	AGTGGGGTGGCTTTTAGGATG
Total HIV DNA and mRNA in primary cells	
CD3OUT5	ACTGACATGGAACAGGGGAAG
CD3OUT3	CCAGCTCTGAAGTAGGGAACATAT
CD3IN5	GGCTATCATTCTTCTTCAAGGT

CD3IN3	CCTCTCTTCAGCCATTTAAGTA
CD3 Taq	LC640AGCAGAGAACAGTTAAGAGCCTCCAT-BBQ
HIV L1	ATGCCACGTAAGCGAAACTCTGGGTCTCTCTDGTTAGAC
HIV R1	CCATCTCTCTCCTTCTAGC
HIV L2	ATGCCACGTAAGCGAAACT
HIV R2	CTGAGGGATCTCTAGTTACC
HIV Taq	LC640-CACTCAAGGCAAGCTTTATTGAGGC-BBQ
Antioxidant gene expression	
GPX1 F	CAACCAGTTTGGGCATCAG
GPX1 R	GTTCACCTCGCACTTCTCG
GPX4 F	GCCTTCCCGTGTAACCAGT
GPX4 R	GCGAACTCTTTGATCTCTTCGT
GSR F	CTTGCGTGAATGTTGGATGT
GSR R	GACCTCTATTGTGGGCTTGG
B2M F	GCCCAAGATAGTTAAGTGGGATCG
B2M R	TCATCCAATCCAAATGCGGC
