# Supplementary material for: Antioxidant nanozyme counteracts HIV‐1 by modulating intracellular redox potential
Source: EMBO Mol Med. 2021 Apr 1;13(5):e13314. doi: 10.15252/emmm.202013314 (PMC8103102; doi:10.15252/emmm.202013314)
Supplement: Supplementary file 2 — Expanded View Figures PDF [file EMMM-13-e13314-s004.pdf]

Expanded View Figures

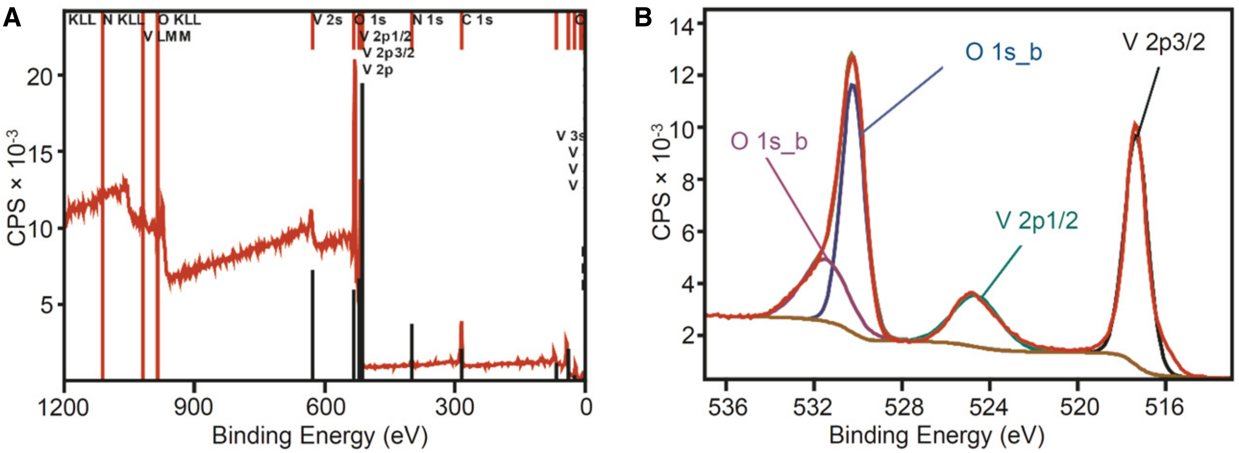

C

V2p3/2	Binding Energy (eV)	517.3451
	FWHM	1.24718
V2p1/2	Binding Energy (eV)	524.6374
	FWHM	2.50811
O1s_a	Binding Energy (eV)	530.2408
	FWHM	1.23586
O1s_b	Binding Energy (eV)	531.5117
	FWHM	2.44992

Figure EV1. X-Ray Photoelectron Spectroscopy (XPS) of Vs.

A, B Left: wide spectra (A) and right: deconvoluted spectra of oxygen and vanadium peaks (B). The spectra were calibrated by taking C1s (284.6 eV) as a standard. As described by *Mendialdua et al* (Mendialdua *et al*, 1995; Hryha *et al*, 2012) the difference in binding energies between the O1s core level and the V2p3/2 level (Δ = BE (O1s) − BE (V2p3/2)) was used to determine the oxidation state of V_2O_5 nanozymes which confirmed + 5 oxidation states of vanadium in Vs.

C Full width half maxima (FWHM) and binding energies of deconvoluted oxygen and vanadium XPS peaks.

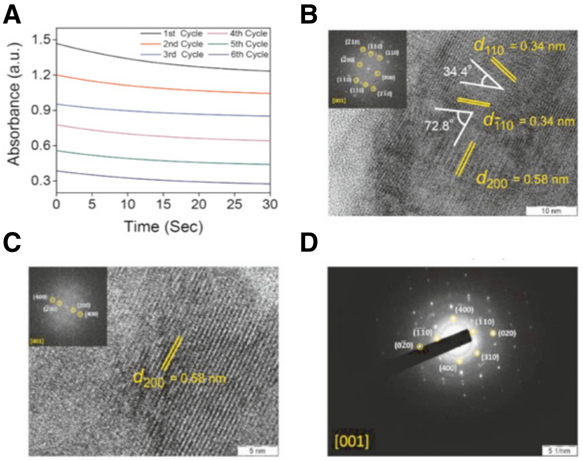

Figure EV2. *In vitro* recycling ability of Vs.

A Recycling activity of Vs during multiple rounds of catalysis was analyzed by addition of fresh substrates in the reaction mixture up to six cycles. The curves observed due to reduction in NADPH absorbance, for every cycle were parallel to each other, indicating no change in their initial rate and persistence of the activity. This clearly depicts robustness of the catalyst. Conditions used for the assay were sodium phosphate buffer (100 mM, pH 7.4), GSH (2 mM), NADPH (0.2 mM), catalyst (20 ng/μl), GR (~1.7 U), and H_2O_2 (20 μM) at 25°C.

B, C High-Resolution TEM (HRTEM) image and FFT patterns of Vs before (B) and after (C) catalysis.

D SAED pattern of Vs after catalysis. The pattern was indexed in a common zone axis [001] which indicates the surface exposed facets are retained after multiple rounds of catalysis.

© 2021 The Authors

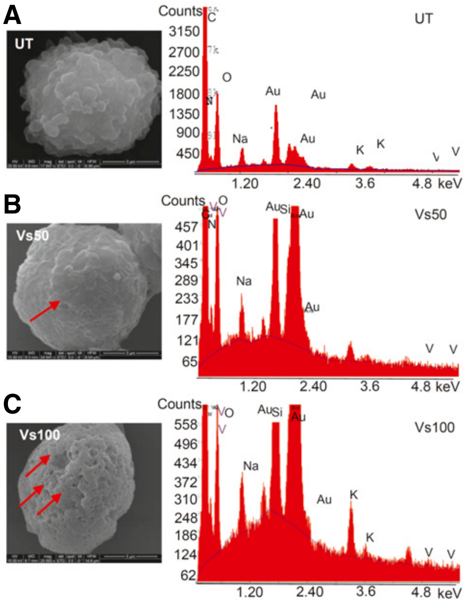

Figure EV3. EDS coupled scanning electron microscopy analysis of Vs-treated U1 cells.

A–C U1 cells were either left untreated (A) or treated with (B) 50 and (C) 100 ng/µl of Vs for 15 min and immediately harvested followed by fixation, dehydration, and imaging by scanning electron microscopy. *Left panels*: The depressions (marked by arrows) formed on the cell surface (B and C). Scale bar 3 µm (A) and (B), 5 µm (C). *Right panels*: The depressions on cell surfaces due to Vs internalization were verified by EDS analysis on the depressed regions. The EDS plots showing the absence or presence of vanadium peak in untreated and Vs-treated U1 cells, respectively.

Source data are available online for this figure.

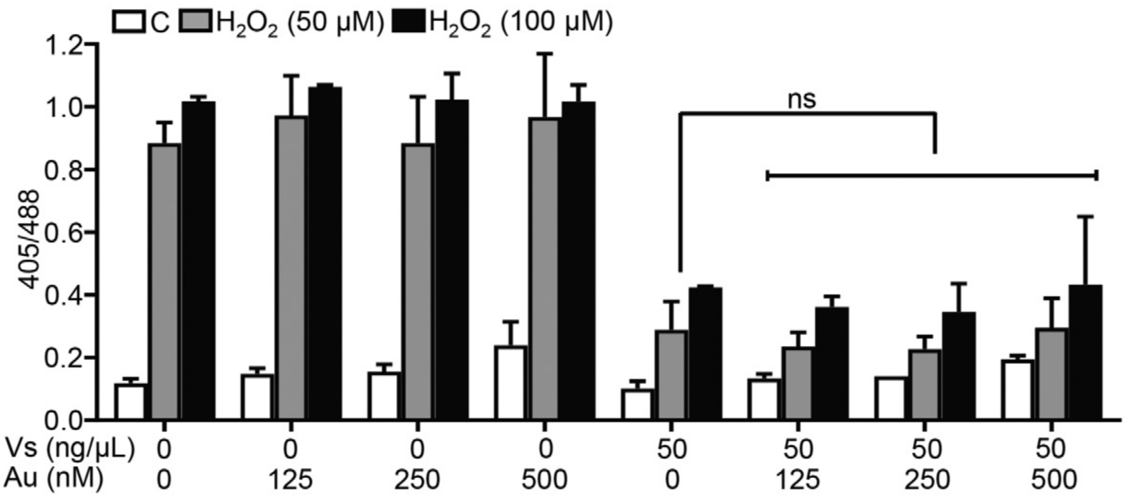

Figure EV4. Auranofin does not influence the antioxidant activity of Vs.

U1 Grx1-roGFP2 cells were either left untreated (C) or supplemented with increasing doses of auranofin for 16 h to inhibit thioredoxin reductase (TrxR). Following this, cells were treated with 50 ng/µl Vs for 15 min and exposed to H_2O_2, and the ratiometric response was measured by flow cytometry. Data are representative of results from two independent experiments performed in duplicate (mean ± SEM). ns—non-significant, by Mann–Whitney Test.

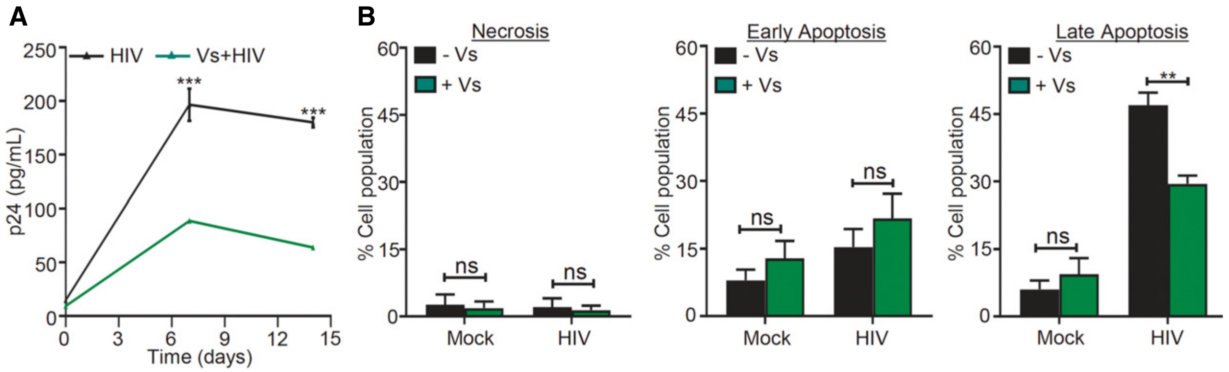

Figure EV5. Vs inhibits HIV-1 replication in HMDMs and reduces late apoptosis in HIV-infected cells.

A Human monocyte-derived macrophages (HMDMs) were pre-treated with 12.5 ng/μl of Vs for 15 min, followed by infection with HIV-1 NL-AD8. Viral release in supernatant was quantified by p24 ELISA at 7 and 14 dpi. Vs treatment was repeated every 72 h. Data are obtained from one healthy donor in duplicate (mean ± SD).

B Survival of HIV-infected primary $CD4^+$ T cells was monitored by Annexin V/PI staining at 3 dpi in presence or absence of Vs treatment. Percentage of necrotic (PI^+), early apoptotic (Annexin V^+), and late apoptotic (Annexin V^+/PI^+) cells were plotted**.** Data are aggregated from three healthy donors (mean ± SEM).

Data information: ***$P < 0.001$, **$P < 0.01$, ns—non-significant analyzed by 2-way ANOVA.

 © 2021 The Authors
