# Supplementary material for: Antioxidant nanozyme counteracts HIV‐1 by modulating intracellular redox potential
Source: EMBO Mol Med. 2021 Apr 1;13(5):e13314. doi: 10.15252/emmm.202013314 (PMC8103102; doi:10.15252/emmm.202013314)

Corresponding to Figure EV3

U1 untreated cells

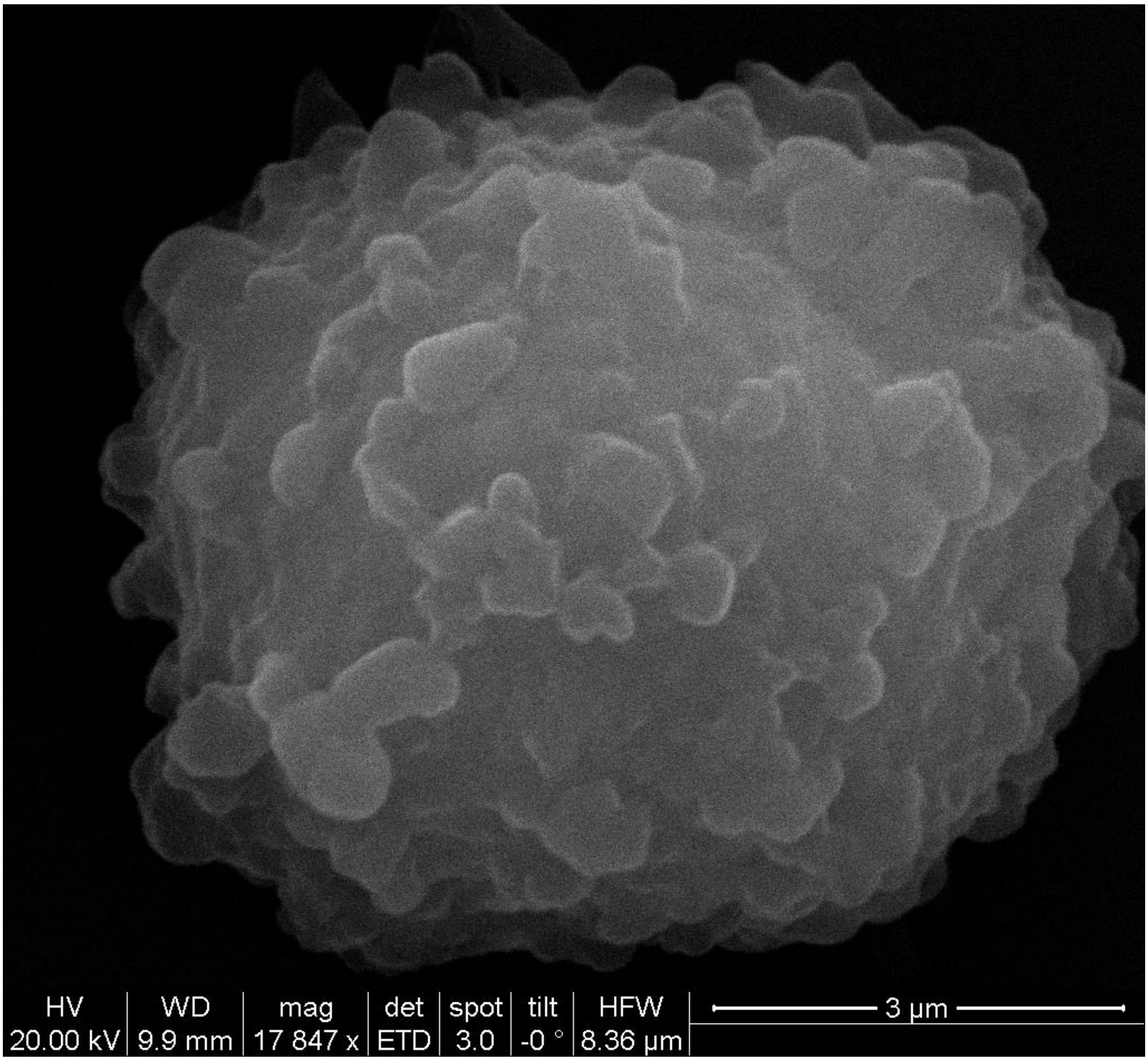

U1 cells treated with 50 ng/µL Vs

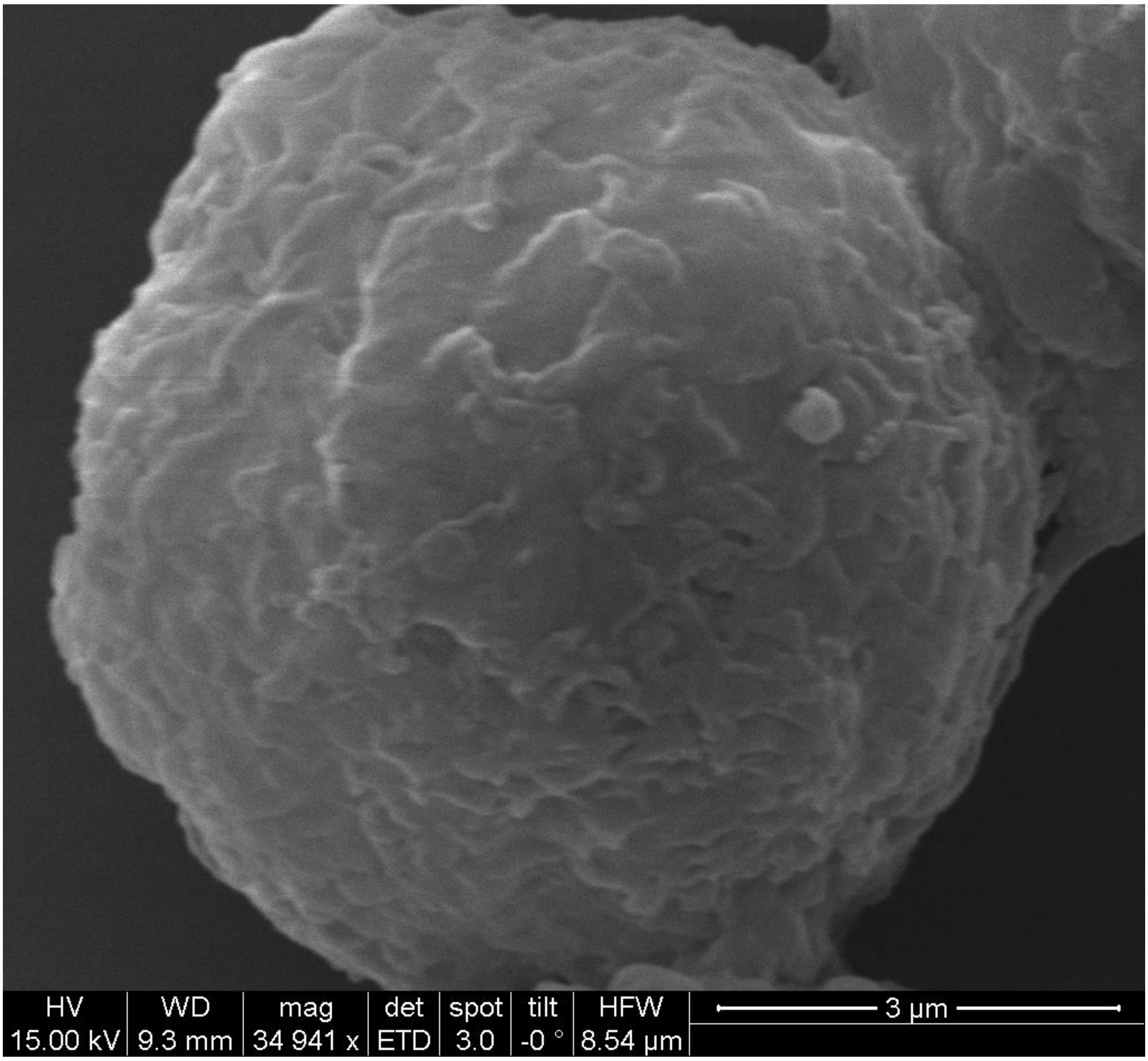

U1 cells treated with 100 ng/µL Vs

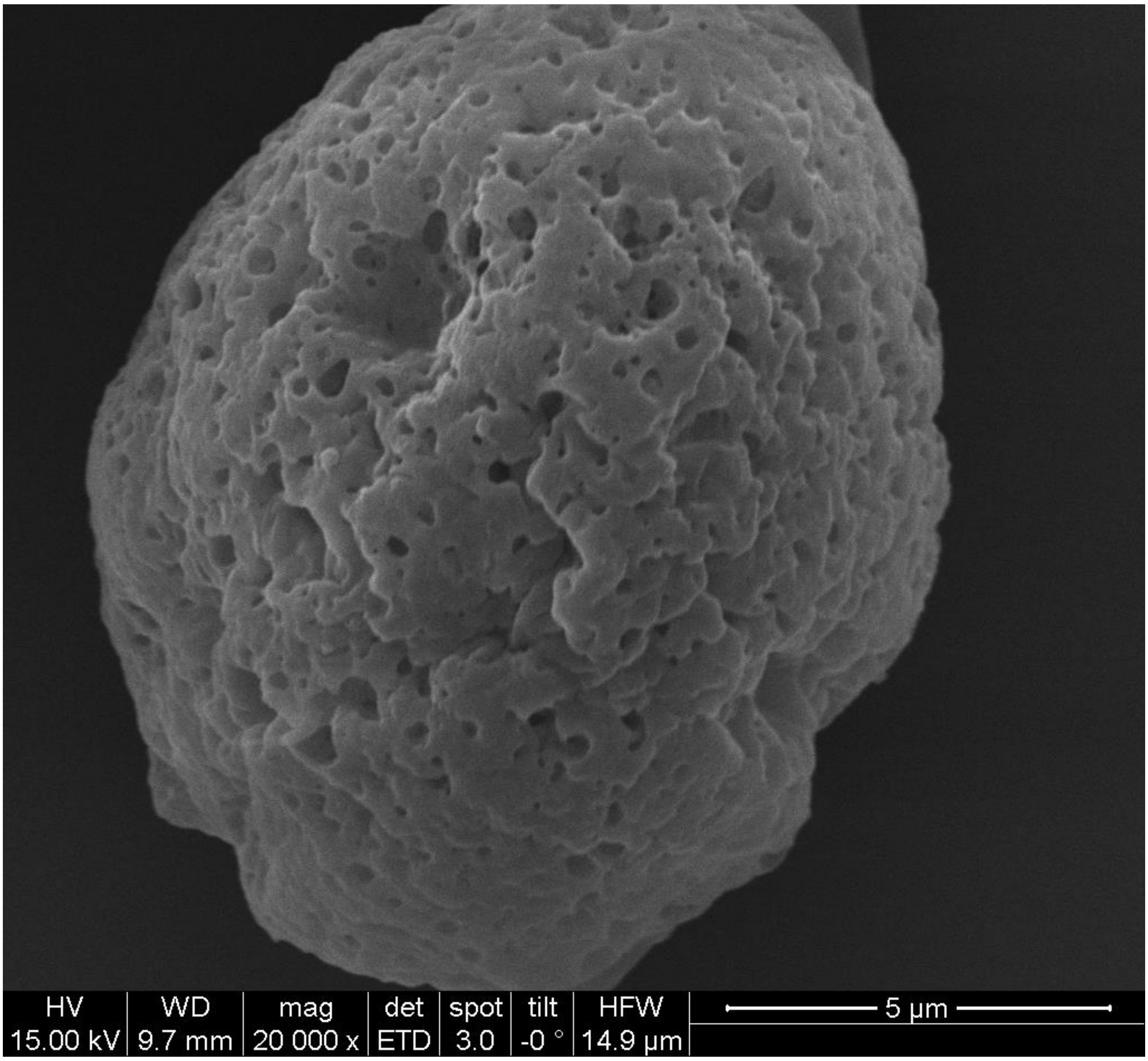

Supplement: Supplementary file 3 — Source Data for Expanded View [file EMMM-13-e13314-s002.zip › emmm202013314-sup-0004-SDataEV/emmm202013314-sup-0004-SDataEV.pdf]
