# Supplementary figures and images for: Antioxidant nanozyme counteracts HIV‐1 by modulating intracellular redox potential
Source: EMBO Mol Med. 2021 Apr 1;13(5):e13314. doi: 10.15252/emmm.202013314 (PMC8103102; doi:10.15252/emmm.202013314)

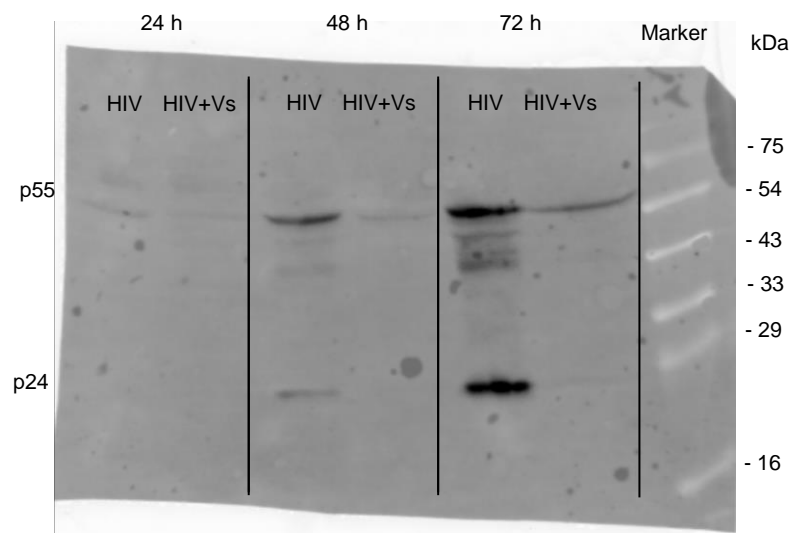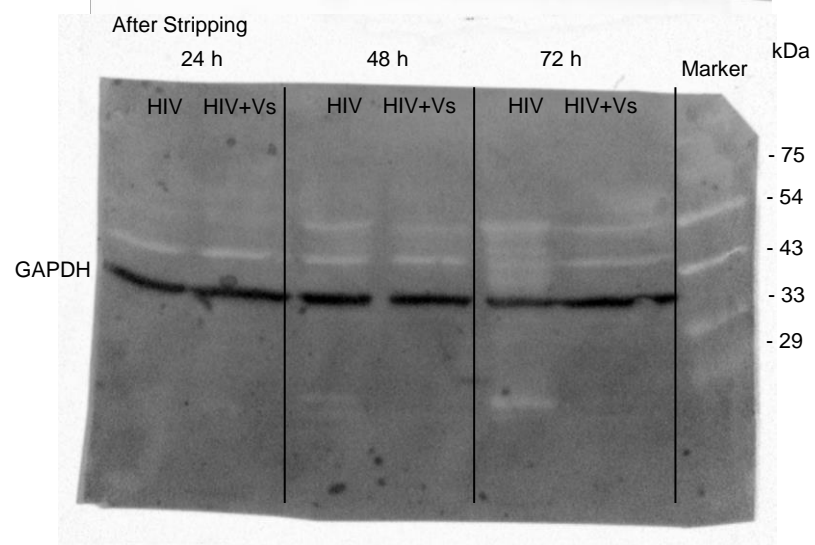

Supplement: Supplementary file 5 — Source Data for Figure 6 [file EMMM-13-e13314-s001.pdf]
